# Single Cell Transcriptomic Analysis in a Mouse Model of Barth Syndrome Reveals Cell-Specific Alterations in Gene Expression and Intercellular Communication

**DOI:** 10.3390/ijms241411594

**Published:** 2023-07-18

**Authors:** Gayani Perera, Liam Power, Amy Larson, Christina J. Codden, Junya Awata, Rebecca Batorsky, Douglas Strathdee, Michael T. Chin

**Affiliations:** 1 Molecular Cardiology Research Institute, Tufts Medical Center, Boston, MA 02111, USA; gayani.perera@tuftsmedicine.org (G.P.); amy.larson.gordon@gmail.com (A.L.); cjcodden@gmail.com (C.J.C.); j_awata@charter.net (J.A.); 2 Medical Scientist Training Program, Tufts University School of Medicine, Boston, MA 02111, USA; liam.power@tufts.edu; 3 Data Intensive Studies Center, Tufts University, Medford, MA 02155, USA; rebecca.batorsky@tufts.edu; 4 Cancer Research UK Beatson Institute, Glasgow G61 1BD, UK; d.strathdee@beatson.gla.ac.uk

**Keywords:** Tafazzin, Barth Syndrome, single-nucleus RNA sequencing, cardiomyopathy, mitochondria, gene expression, metabolism

## Abstract

Barth Syndrome, a rare X-linked disorder affecting 1:300,000 live births, results from defects in Tafazzin, an acyltransferase that remodels cardiolipin and is essential for mitochondrial respiration. Barth Syndrome patients develop cardiomyopathy, muscular hypotonia and cyclic neutropenia during childhood, rarely surviving to middle age. At present, no effective therapy exists, and downstream transcriptional effects of Tafazzin dysfunction are incompletely understood. To identify novel, cell-specific, pathological pathways that mediate heart dysfunction, we performed single-nucleus RNA-sequencing (snRNA-seq) on wild-type (WT) and *Tafazzin*-knockout (Taz-KO) mouse hearts. We determined differentially expressed genes (DEGs) and inferred predicted cell–cell communication networks from these data. Surprisingly, DEGs were distributed heterogeneously across the cell types, with fibroblasts, cardiomyocytes, endothelial cells, macrophages, adipocytes and pericytes exhibiting the greatest number of DEGs between genotypes. One differentially expressed gene was detected for the lymphatic endothelial and mesothelial cell types, while no significant DEGs were found in the lymphocytes. A Gene Ontology (GO) analysis of these DEGs showed cell-specific effects on biological processes such as fatty acid metabolism in adipocytes and cardiomyocytes, increased translation in cardiomyocytes, endothelial cells and fibroblasts, in addition to other cell-specific processes. Analysis of ligand–receptor pair expression, to infer intercellular communication patterns, revealed the strongest dysregulated communication involved adipocytes and cardiomyocytes. For the knockout hearts, there was a strong loss of ligand–receptor pair expression involving adipocytes, and cardiomyocyte expression of ligand–receptor pairs underwent reorganization. These findings suggest that adipocyte and cardiomyocyte mitochondria may be most sensitive to mitochondrial Tafazzin deficiency and that rescuing adipocyte mitochondrial dysfunction, in addition to cardiomyocyte mitochondrial dysfunction, may provide therapeutic benefit in Barth Syndrome patients.

## 1. Introduction

Barth Syndrome (BTHS), a rare X-linked mitochondrial disorder affecting 1:300,000 live births, is characterized by childhood onset cardiomyopathy, skeletal myopathy, cyclic neutropenia and premature death. To date, no efficacious therapy exists. The affected gene encodes Tafazzin, an inner mitochondrial membrane-associated transacylase and critical regulator of mitochondrial membrane composition. Tafazzin remodels the mitochondrial membrane phospholipid, cardiolipin, from its immature monolyso-isoform (MLCL) to its mature isoform (CL). At the organellar level, mitochondria lacking functional Tafazzin display altered cristae structure, reduced oxygen consumption, increased reactive oxygen species generation, altered substrate utilization and an elevated MLCL to CL ratio (reviewed in [1,2,3]). Failure to remodel cardiolipin to its mature form affects mitochondrial inner membrane curvature, resulting in a failure to form electron transport protein supercomplexes [4,5]. Disease-causing mutations in Tafazzin are present throughout the molecule and can affect protein localization, protein stability and enzymatic activity [6,7,8,9]. Although genomically encoded, Tafazzin is transported across the outer mitochondrial membrane and then localized to the inner mitochondrial membrane, directed by specific peptide domains [10,11]. The molecular, cellular, tissue, organ and physiological mechanisms by which Tafazzin mutations propagate dysfunction across multiscale networks are poorly understood, and a deeper understanding of the components of these networks will likely provide fundamental advances in knowledge of mitochondrially associated disease mechanisms and the development of targeted therapies for mitochondrial disorders such as BTHS. 

Single-cell RNA sequencing methods have facilitated the analysis of cell-specific gene expression, cellular diversity and intercellular communication in a wide variety of tissues and across a wide variety of species—including human and mouse hearts—for both normal and diseased conditions [12,13,14,15,16,17,18,19,20,21]. As a first step in identifying transcriptional networks and pathological pathways that mediate the effects of Tafazzin deficiency in the heart at the single-cell level, we have performed single-nuclei transcriptomics on heart tissue extracted from a *Tafazzin* knockout (Taz-KO) mouse model of Barth Syndrome [22]. We have identified differentially expressed genes by genotype for specific cell populations and have found that fibroblasts, cardiomyocytes, endothelial cells, macrophages, adipocytes and pericytes demonstrate the largest numbers of DEGs. We correlated these cell-specific DEGs with potentially important biological processes through a Gene Ontology (GO) analysis and found cell-specific perturbations in metabolic pathways. We also analyzed ligand–receptor (L-R) pair gene expression to infer intercellular communication networks and identified extensive alterations in potential communication patterns, notably involving adipocytes.

## 2. Results

### 2.1. Tafazzin Deficiency Is Not Associated with a Disease-Specific Cell Population in the Heart

**Figure 1:**
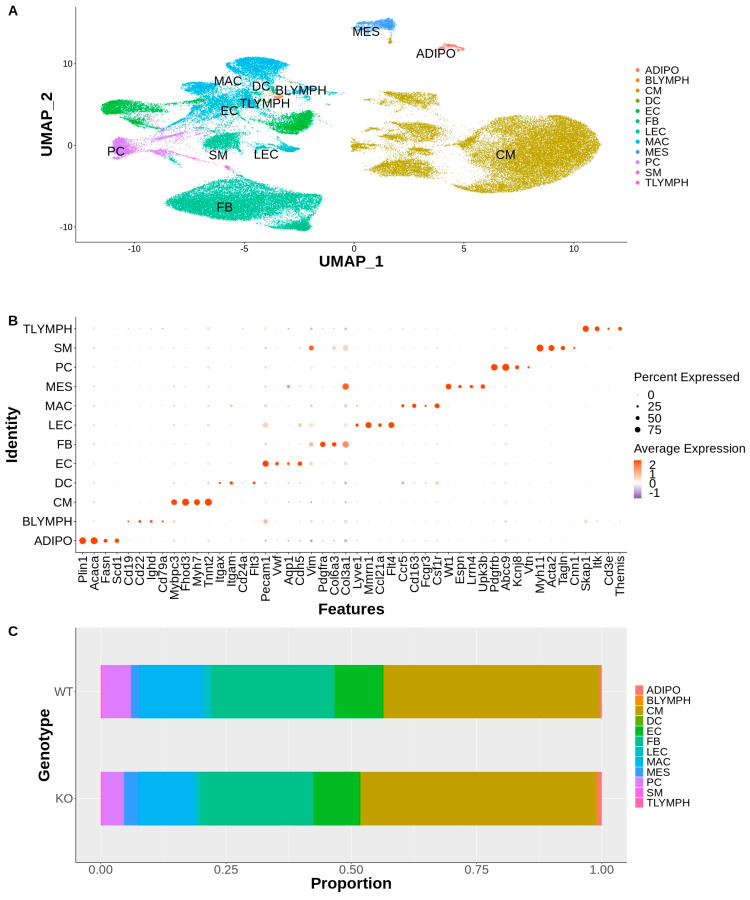
Single-nucleus RNA sequencing of wild-type and *Tafazzin*-knockout hearts reveals diverse cell types but no genotype-specific cell population or differences in distribution of major cell types: (**A**) UMAP plot of major cell types of the heart identified in the dataset. Each dot represents a single nucleus colored by its cell type identity. (**B**) Dot plot showing expression of known cell markers for identified cell types. (**C**) Distribution of cell types for each genotype.

Whole hearts from four male WT and four male KO mice were used to generate the snRNA-seq dataset [17,18,19,20]. To identify the major cell types present in our data, we performed cell and gene-level quality control, followed by sample integration and graph-based clustering as described in Materials and Methods [23,24,25,26,27,28,29,30]. The integrated dataset, consisting of the transcriptomes from 75,051 nuclei, revealed a total of 35 distinct clusters, represented by the Uniform Manifold Approximation and Projection (UMAP) plot shown in Supplemental Figure S1. The 35 clusters represented 12 major cell types found in the heart (Figure 1A,B).

Analysis of the distribution of these cell types per genotype revealed that all major cell types were represented by each genotype and no disease-specific cell clusters were present (Supplemental Figure S2). There were no significant differences between genotypes in the cell type distribution (Figure 1C), as determined by calculating the cell type diversity statistic shown in Supplemental Figure S3 (*t*-test *p*-value 0.66) [31]. The nuclei counts for each cell type and genotype is listed in Supplemental Table S1.

Major cell types identified include:

- Adipocytes (ADIPOs);
- B-Lymphocytes (BLYMPHs);
- Cardiomyocytes (CMs);
- Dendritic Cells (DCs);
- Endothelial Cells (ECs);
- Fibroblasts (FBs);
- Lymphatic Endothelial Cells (LECs);
- Macrophages (MACs);
- Mesothelial Cells (MESs);
- Pericytes (PCs);
- Smooth Muscle Cells (SMs);
- T-Lymphocytes (TLYMPHs).

### 2.2. Tafazzin Deficiency Is Associated with Differential Expression of Genes That Varies by Cell Type and Is Associated with Alterations in Multiple Biological Processes

We performed differential expression testing between Taz-KO and WT cells within each cell type using a generalized linear model framework as described in Materials and Methods [26,27,28,29,32]. Genes were considered to be differentially expressed if the absolute value of their log_2_fold-change was greater than 0.58 (adjusted *p*-value < 0.001). Surprisingly, DEGs were distributed heterogeneously, with fibroblasts, cardiomyocytes, endothelial cells, macrophages, adipocytes and pericytes exhibiting the greatest number of DEGs between genotypes; the lymphatic endothelial and mesothelial cell types each only had one differentially expressed gene detected, and none were detected in the lymphocytes, dendritic cells or smooth muscle cells (Table 1). The full list of DEGs for each cell type are listed in Supplemental Table S2. 

**Figure 2:**
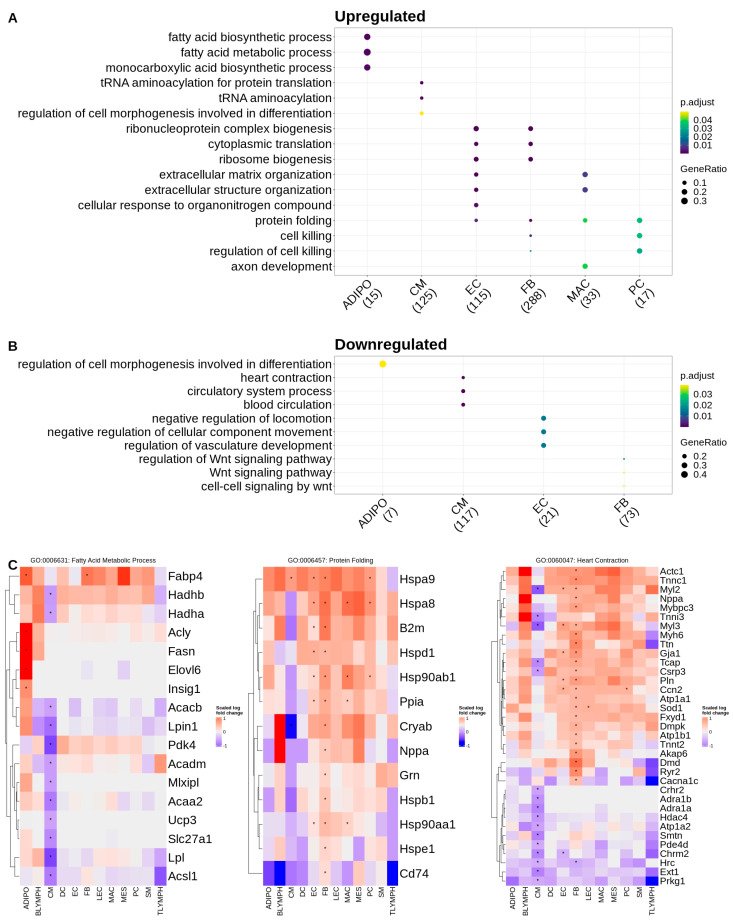
A Gene Ontology analysis of differentially expressed genes reveals cell-specific differences in multiple biological processes associated with Tafazzin deficiency: (**A**) Dot plot of over-represented biological process terms, by cell type, for genes upregulated in Taz-KO cells. Only the top three terms, by count for each cell type, are displayed. (**B**) Dot plot of over-represented biological process terms, by cell type, for genes downregulated in Taz-KO cells. Only the top three terms, by count for each cell type, are displayed. (**C**) Heatmaps showing scaled expression of genes associated with select over-represented ontology terms. Asterisks indicate differentially expressed genes with adjusted *p*-values < 0.001 and |average log2 fold change| > 0.58.

To further understand the biological context of these DEGs and identify potential cell-specific dysregulated pathways associated with Tafazzin deficiency, we also performed a Gene Ontology over-representation test for biological process terms on the sets of upregulated (increased expression in Taz-KO) and downregulated (decreased expression in Taz-KO) DEGs within each cell type. Over-represented terms for upregulated genes in the adipocytes largely involved lipid metabolic processes, such as acetyl-CoA and fatty acid synthesis, and were driven by the genes *Acly*, *Fasn*, *Insig1* and *Elovl6*. Interestingly, fatty acid metabolism and specifically fatty acid beta-oxidation mapping genes such as *Hadhb*, *Hadha*, *Acaa2*, *Acacb* and *Acadm* were downregulated in cardiomyocytes. Downregulated genes in adipocytes mapped only to one term—regulation of cell morphogenesis involved in differentiation—which was again conversely upregulated in the cardiomyocytes. Other terms mapping to the upregulated genes in cardiomyocytes included tRNA aminoacylation and were driven by the upregulation of aminoacyl-tRNA synthetase genes. Downregulated genes in the cardiomyocytes also mapped to the circulatory system and muscle-contraction-related terms. Upregulated DEGs in the endothelial and fibroblast cell types mapped to the greatest number of Gene Ontology terms among the various cell types. As with the cardiomyocytes, upregulated terms for the fibroblasts and endothelial cells included those related to protein translation such as ribosome biogenesis. Regulation of vasculature development and Wnt signaling processes were downregulated in the endothelial cells and fibroblasts, respectively. The upregulated gene set for macrophages mapped to extracellular matrix organization terms, and in pericytes, the upregulated genes mapped to the regulation of cell killing and protein folding, with both terms being driven by heat shock family proteins such as *Hsp90ab1* and *Hspa8*. Zero terms mapped to the downregulated gene set in pericytes. Protein folding terms were common to the upregulated gene sets of fibroblasts, macrophages, and endothelial cells in addition to pericytes (Figure 2). The full list of GO terms over-represented by gene sets that are upregulated and downregulated for each cell type are listed in Supplemental Table S3. 

### 2.3. Tafazzin Deficiency Is Associated with Alterations in General and Cell-Specific Ligand–Receptor Pair Gene Expression

**Figure 3:**
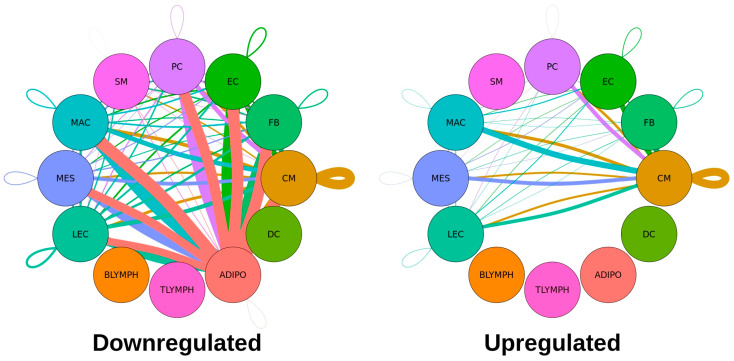
A ligand–receptor pair expression analysis reveals downregulation of many predicted cell–cell interactions in Taz-KO cells. The number of significantly dysregulated ligand–receptor interactions between two cell types are graphically represented by the thickness of lines connecting two circles (cell types) and the color of a line reflects the cell type from which the ligands are being broadcasted from. Dysregulated communication that is downregulated in the Taz-KO cells are shown on the (**left**) and dysregulated communication that is upregulated in the Taz-KO cells are shown on the (**right**).

To determine how Tafazzin deficiency affects potential communication between the major cell types, we performed a ligand–receptor (L-R) pair gene expression analysis comparing the two genotypes, as described in Materials and Methods [33]. Through this analysis, 9011 dysregulated pairs were identified, with 7237 pairs downregulated and 1774 pairs upregulated in the Taz-KO condition. A tabular listing of the numbers of dysregulated ligands and receptors for each cell type is shown in Table 2. A graphical representation of these significantly dysregulated L-R interactions, separated by upregulation or downregulation in the Taz-KO cells, are shown in Figure 3.

Notably, adipocytes were associated with the largest number of dysregulated ligand–receptor interactions, and all significant pairs involving this cell type—as either the broadcasting or receiving cell type—were downregulated, suggesting a major disruption of cell–cell signaling involving adipocytes occurs among Tafazzin-deficient cells. Cardiomyocytes expressed the second largest number of dysregulated L-R pairs. For the remaining cell types with significantly dysregulated communication, this pattern of more downregulated than upregulated L-R pairs held, and there were no significant interactions involving B-lymphocytes, T-lymphocytes or dendritic cells. 

**Figure 4:**
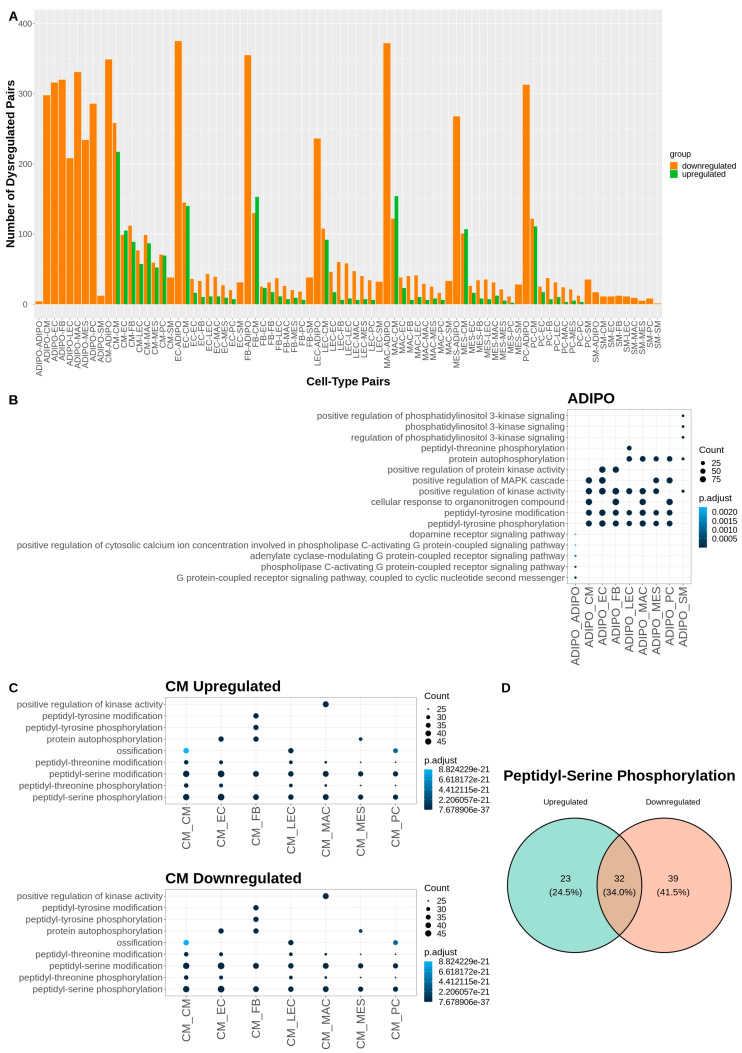
A biological process Gene Ontology analysis of dysregulated ligand–receptor pairs reveals differences in communication associated with Tafazzin deficiency: (**A**) Number of dysregulated pairs between two communicating cell types. (**B**) Dot plot of over-represented biological process terms, by cell type, for all genes contributing to dysregulated signaling involving adipocytes. Only the top three terms, by count for each cell type pair, are displayed. (**C**) Dot plot of over-represented biological process terms, by cell type, for all genes contributing to up or downregulated signaling involving cardiomyocytes. Only the top 3 terms, by count for each cell type pair, are displayed. (**D**) Venn diagram showing overlap of genes of dysregulated ligand–receptor pairs that contribute to the GO term “Peptidyl-Serine Phosphorylation” in up- and downregulated L-R pairs involving cardiomyocytes.

Given that the major shares of dysregulated communication involve the adipocyte and cardiomyocyte cell types (Figure 4A), we performed another Gene Ontology over-representation analysis on the dysregulated ligands and receptors for each of the communicating cell type pairs that included adipocytes or cardiomyocytes (Figure 4). For adipocyte signaling, there was a broad downregulation of kinase activity and peptidyl-tyrosine phosphorylation with CM, EC, FB, LEC, MAC, MES and PC cells. Phospholipase C activation signaling was downregulated in adipocyte communication with other adipocytes and PI3K communication was downregulated between adipocytes and smooth muscle cells (Figure 4B). Interestingly, for cardiomyocyte signaling, the GO analysis revealed both a broad upregulation and downregulation of peptidyl-serine and peptidyl-threonine phosphorylation. This concordance among terms represented by opposing upregulated and downregulated signaling L-R pairs may be attributable to the broader nature of these biological process terms. For example, when looking at the specific up- and downregulated gene sets that contributed to the GO term “Peptidyl-Serine Phosphorylation”, there was only a 34% overlap in the contributing gene lists (Figure 4C,D). Other cell-pair-specific ontology terms associated with cardiomyocyte signaling included upregulation of cell growth driven by signaling between CMs and FBs, upregulation of muscle cell proliferation driven by CM and MAC signaling as well as downregulation of ossification driven by communication between cardiomyocytes and LECs, PCs and other CMs (Figure 4C). A table of all significantly dysregulated L-R pairs can be found in Supplemental Table S4.

## 3. Discussion

Barth Syndrome has long been known to result from mitochondrial dysfunction due to abnormal Tafazzin-dependent cardiolipin remodeling in the inner mitochondrial membrane that alters the efficiency of mitochondrial respiration (reviewed in [1,2,3]). Clinically, the disease manifests primarily in the heart, skeletal muscle and blood through the development of cardiomyopathy, skeletal myopathy and cyclic neutropenia. Since mitochondria are present in all cells, the underlying susceptibility of specific cell types to dysfunction associated with Tafazzin deficiency has not been established—although the effects on striated muscle have been assumed to occur from the high energy requirements and increased mitochondrial numbers associated with these tissues. Here we report the first single-nucleus transcriptomic analysis of hearts from *Tafazzin*-knockout mice that model Barth Syndrome [22,34]. As expected, Tafazzin loss of function is associated with widespread changes in cardiomyocyte gene expression, but other observed changes in adipocytes, fibroblasts, endothelial cells, macrophages and pericytes were not anticipated. The lack of expression differences among the other immune cell types may indicate these cells are less involved in the early-stage phenotype of this mouse model of BTHS. Analysis of predicted cell–cell communication networks through modeling ligand–receptor pair gene expression was notable for a marked downregulation of cell–cell interactions across all cell types in the Taz-KO condition. Given the sensitivity of this cell–cell communication analysis to differences in the product of a ligand and receptor’s expression across many cell type pairs, the contrast in the number of identified dysregulated L-R pairs compared to the number of identified DEGs per individual cell type may be expected. The predicted loss of cell–cell communication has also been noted in other conditions such as hypertrophic cardiomyopathy [17,19,20]. 

In a healthy heart, fatty acid metabolism serves as the primary pathway through which ATP levels, and therefore the heart’s contractile ability, is sustained; alterations in energy metabolism, and specifically fatty acid metabolism, have been observed in association with heart failure [35]. Prior studies have shown that in young BTHS patients myocardial fatty acid extraction and uptake is significantly reduced [36]. Here we report that in a mouse model of BTHS, alterations in fatty acid metabolism in the heart are cell type specific. Genes associated with fatty acid synthesis, such as those encoding the acetyl-CoA synthesis enzyme *Acly*, the fatty acid synthase *Fasn* and the fatty acid elongase *Elovl6*, have increased expression in Taz-KO adipocytes, while those associated with fatty acid beta oxidation enzymes such as *Hadhb*, *Hadha*, *Acaa2*, *Acacb* and *Acadm* are downregulated specifically in KO cardiomyocytes. Proteomic studies of cardiac mitochondria isolated from WT and Taz-shRNA-knockdown mice have implicated dysregulation of CoA-dependent fatty acid metabolism in BTHS through the downregulation of multiple enzymes involved in fatty acid oxidation [37]. We observed some concordance with this study in downregulation of CoA metabolism genes such as the medium-chain acyl-CoA dehydrogenase *Acadm* and the long-chain acyl-CoA synthetase *Acsl1* in our Taz-KO cardiomyocytes [37]. These findings may also be relevant to the reduced adiposity seen in this mouse model [22]. The significance of the observed increase across many of the other cell types in expression of genes relating to protein translation and folding is unclear but may be related to a mitochondrial and protein homeostatic stress response in the Tafazzin-deficient cells [38,39,40,41]. The common over-representation of post-translational modification and kinase activity Gene Ontology terms associated with dysregulated ligand–receptor pairs suggest phosphoproteomic studies of this BTHS model may also provide further insight to the biological pathways that are disrupted because of Tafazzin deficiency. 

While single-nuclei transcriptomics is a powerful tool to detect cell-specific differences in expression, the transcriptomes captured from these mice do not provide information on how diverse cell types may be interacting spatially within the context of this disease. This limitation emphasizes the need for further in vitro experiments to validate these results—especially the inferred dysregulation of cell-to-cell communication where the proximity of two cells influences their ligand–receptor interactions. Another potential limitation of this study includes the low sample size of mice. Another limitation of the study is that the age of mice used predates the expected relatively late onset of cardiac impairment in these mice [22,34] and thus the data in this study may not capture some essential pathological cardiac pathways that promote cardiomyopathy at later ages.

## 4. Materials and Methods

### 4.1. Generation of Single-Nuclei RNA-Seq Datasets from Tafazzin Knockout Mouse Hearts

Whole hearts from four male WT and four male Taz-KO mice were minced, cryopreserved and then processed for snRNA-seq library generation and next-generation sequencing as described previously [17,18,19,20] using commercially available reagents (10× Genomics). The Tafazzin-knockout mouse used in this study has been described previously [22,34]. All libraries were generated with tissue from 9-week-old mice. Approximately 10,000 nuclei from each mouse heart were used to generate each mouse heart library. 

### 4.2. Clustering of Cells by Gene Expression Pattern, Assignment of Cell Type Identity, and Determination of Cell Type Distribution

Sequencing reads, including intronic, were processed using Cell Ranger version 6.0.1 using the mm10-2020-A reference transcriptome [23]. Quality control measures were performed to correct for ambient RNA contamination (SoupX version 1.6.2) [24] and reduce doublets (DoubletFinder version 2.0.3) [25], followed by gene and cell-level filtering. When correcting for ambient RNA contamination, empty droplets with 2–10 transcripts (UMIs) were used to estimate the background expression profile and 0.2 was added to the estimated contamination fraction. For gene-level quality control, genes detected in fewer than 10 cells and all genes mapping to the mitochondrial genome were removed. Additionally, cells with fewer than 800 transcripts (nCount_RNA) or fewer than 250 genes (nFeature_RNA) were removed. A total of 75,051 nuclei were used for downstream analyses. Normalization, selection of the top 2000 highly variable genes, scaling and linear dimension reduction (PCA) were performed on a merged dataset using the R package Seurat 4.3.0 [26,27,28,29]. To enable the identification of shared cell types across cells from different genotypes, Harmony version 0.1.0 was used to generate a common reduced dimensional embedding from the merged dataset [30], followed by running Seurat’s UMAP, Nearest Neighbor and clustering functions using the first 15 principal components from these data [26,27,28,29]. A clustering resolution of 0.9 was chosen using the subsampling-based approach ChooseR [42]. The expression of known cell-specific gene markers was used to identify major cell types, as has been done previously [14,16,17,18,19]. To further refine cell assignments, such as distinguishing specific myeloid or lymphoid cell populations, Seurat’s FindSubCluster function was utilized [26,27,28,29]. To determine whether the cell type distribution varied between WT and Taz-KO hearts, we calculated the Cell Type Diversity Statistic [31]. 

### 4.3. Differential Expression Analysis

Differentially expressed genes between WT and Taz-KO cells, for each cell type, were determined using MAST, a generalized linear model framework that uses the proportion of genes expressed in a single cell as a covariate to account for both technical and biological sources of variation [32]. The MAST framework was implemented through Seurat’s FindMarkers function, adjusting for individual mouse variation using the “latent.vars” argument [26,27,28,29]. Genes were considered differentially expressed if the absolute value of their log_2_fold-change was greater than 0.58 (adjusted *p*-value < 0.001). A biological process Gene Ontology analysis was conducted for each set of upregulated and downregualted DEGs, for each cell type, using the compareCluster function of clusterProfiler version 3.18.1 [43]. All genes that passed gene-level quality control filtering were used for the background set (“universe” argument). 

### 4.4. Cell–Cell Communication Analysis

To identify potential cardiac cell–cell communication differences between WT and Taz-KO mouse hearts, we used scLR, a statistical method for examining dysregulated L-R interactions, between two conditions, that models the distribution of ligand and receptor expression and accounts for intersample variance and small sample size [33]. The curated set of L-R pairs used for comparison was obtained from the Omnipath database [44]. Cell communication networks were plotted using igraph version 1.3.5 [45]. Lines in our cell networks connect two cell types (circles) and represent statistically significant dysregulated ligand–receptor pairs (i.e., cell–cell communication between a broadcasting (ligand) and recipient (receptor) cell types). Line color in our networks represents the broadcasting ligand source cell type. Line thickness is proportional to the number of dysregulated ligand–receptor pairs associated between two communicating cell types. The Gene Ontology analysis of differentially expressed ligand–receptor pairs involving adipocytes or cardiomyocytes was performed using the enrichGO function of clusterProfiler [43]. 

## 5. Conclusions

Barth Syndrome cardiomyopathy has been associated with profound alterations in mitochondrial and contractile function, but determination of cellular mechanisms that mediate the effects of Tafazzin deficiency beyond mitochondrial dysfunction and the contribution of nonmyocytes to the cardiomyopathic phenotype have not been fully elucidated. Here, we report that Tafazzin loss of function in a mouse model of Barth Syndrome results in distinct alterations in cardiomyocyte gene expression, and we also identify adipocyte dysfunction as a potential contributor to Barth Syndrome cardiomyopathy, potentially through dysregulated fatty acid metabolism. 

## Figures and Tables

**Table 1:** Number of differentially expressed genes detected in each cell type.

Cell Type	Upregulated	Downregulated	Total
Adipocyte	16	8	24
B-Lymphocyte	0	0	0
Cardiomyocyte	154	141	295
Dendritic	0	0	0
Endothelial	121	21	142
Fibroblast	296	75	371
Lymphatic Endothelial	1	0	1
Macrophage	33	0	33
Mesothelial	1	0	1
Pericyte	17	5	22
Smooth Muscle	0	0	0
T-Lymphocyte	0	0	0

Number of significantly up- or downregulated genes in Taz-KO cells with adjusted *p*-values < 0.001 and |average log_2_fold-change| > 0.58.

**Table 2:** Number of significantly dysregulated ligands and receptors by cell type.

	Downregulated		Upregulated	
Cell Type	Ligands	Receptors	Ligands	Receptors
Adipocyte	2009	2289	0	0
B-Lymphocyte	0	0	0	0
Cardiomyocyte	1162	1295	676	974
Dendritic	0	0	0	0
Endothelial	749	622	204	217
Fibroblast	680	679	226	143
Lymphatic Endothelial	661	541	142	114
Macrophage	716	635	213	132
Mesothelial	555	452	157	95
Pericyte	620	476	156	99
Smooth Muscle	85	248	0	0
T-Lymphocyte	0	0	0	0
Total	7237	7237	1774	1774

## Data Availability

The datasets used in this study are available online via the Gene Expression Omnibus database under accession number GSE235047.

## Supplementary Materials

The following supporting information can be downloaded at: https://www.mdpi.com/article/10.3390/ijms241411594/s1.
