# Supplementary material for: Single Cell Transcriptomic Analysis in a Mouse Model of Barth Syndrome Reveals Cell-Specific Alterations in Gene Expression and Intercellular Communication
Source: Int J Mol Sci. 2023 Jul 18;24(14):11594. doi: 10.3390/ijms241411594 (PMC10380964; doi:10.3390/ijms241411594)
Supplement: Supplementary file 1 [file ijms-24-11594-s001.zip › ijms-2491397-supplementary/Supplemental Figures_R1(GP.edits)_07132023.pptx]

## Slide 1
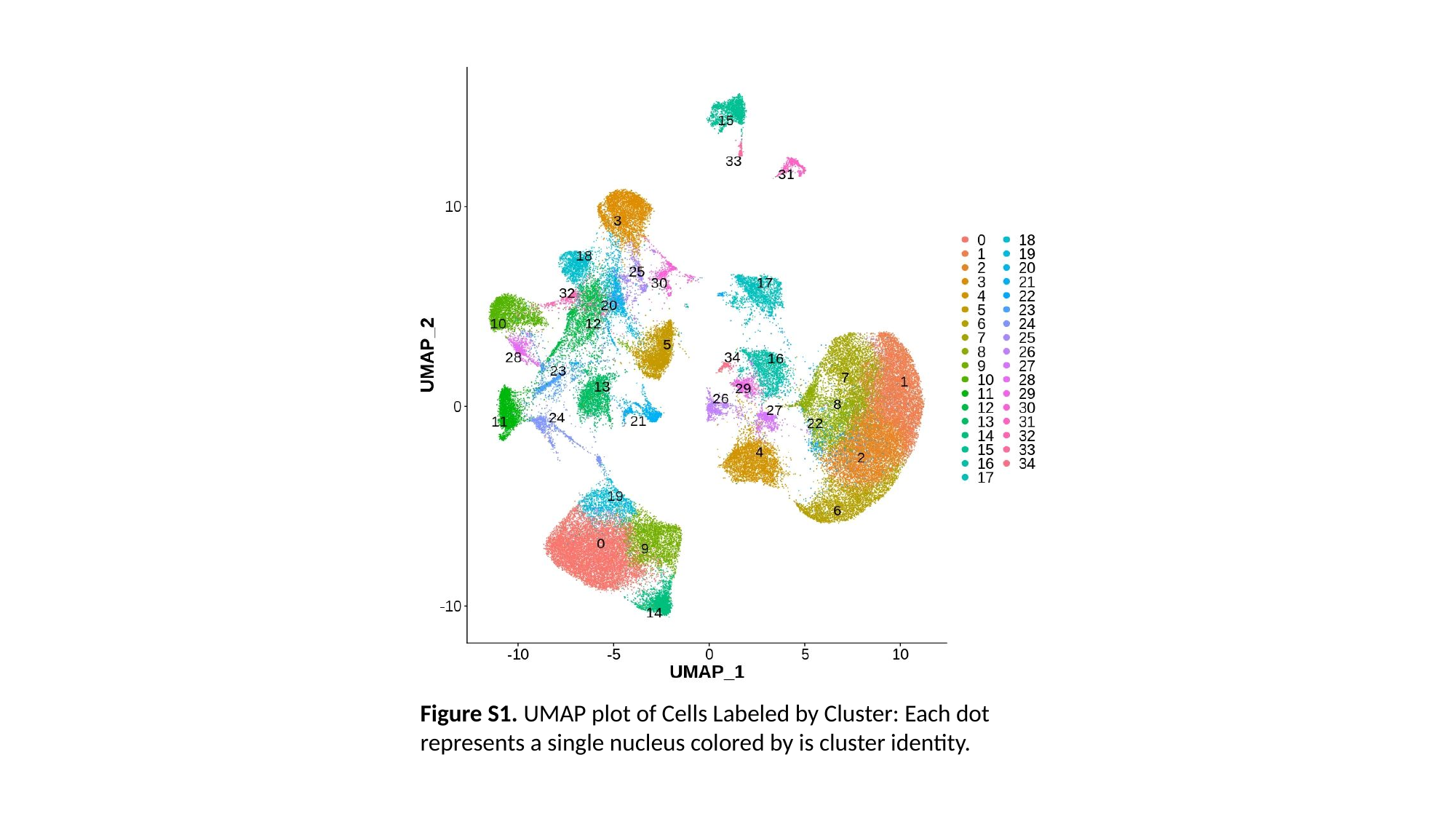

Figure S1. UMAP plot of Cells Labeled by Cluster: Each dot represents a single nucleus colored by is cluster identity.

## Slide 2
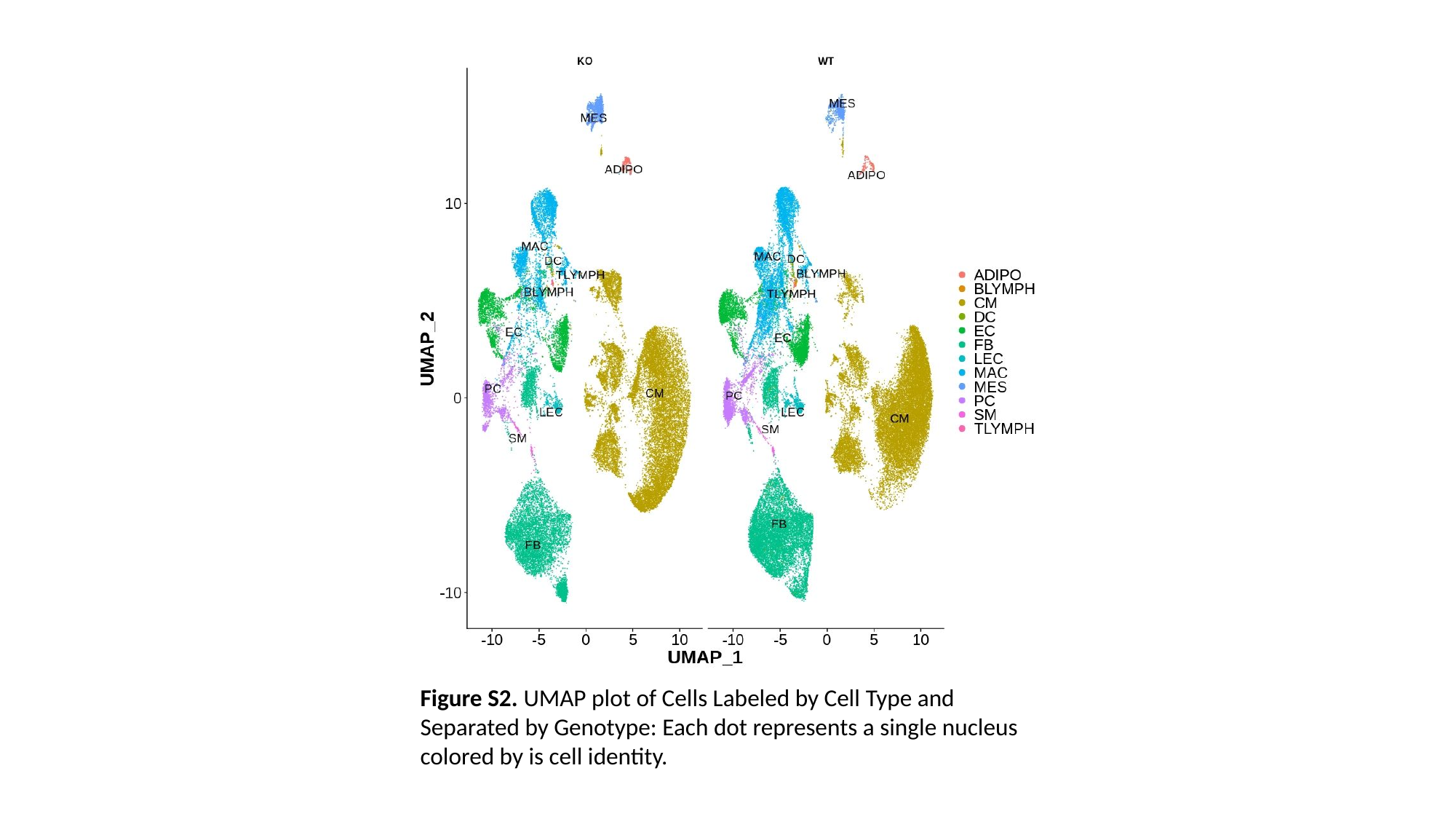

Figure S2. UMAP plot of Cells Labeled by Cell Type and Separated by Genotype: Each dot represents a single nucleus colored by is cell identity.

## Slide 3
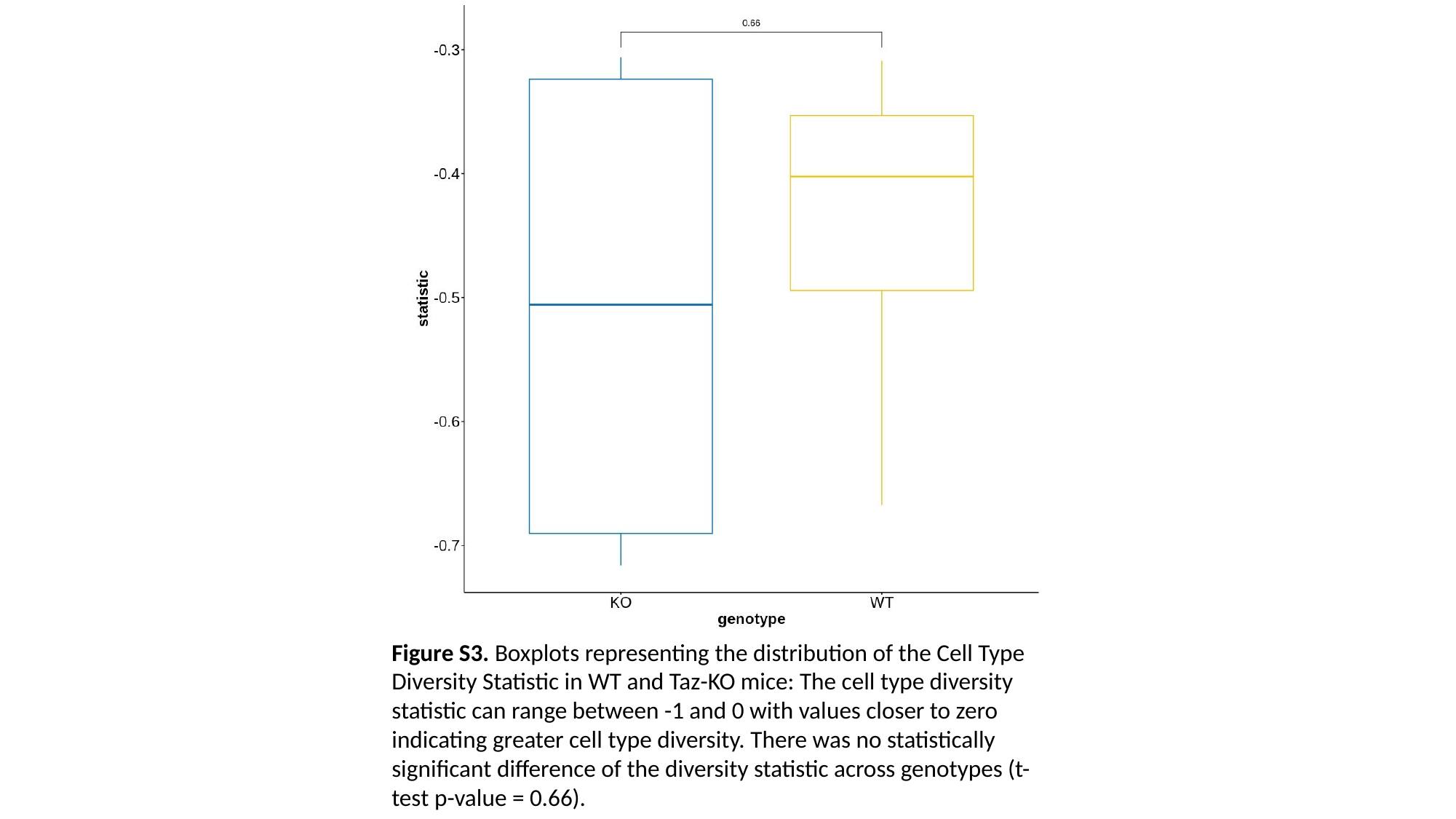

Figure S3. Boxplots representing the distribution of the Cell Type Diversity Statistic in WT and Taz-KO mice: The cell type diversity statistic can range between -1 and 0 with values closer to zero indicating greater cell type diversity. There was no statistically significant difference of the diversity statistic across genotypes (t-test p-value = 0.66).
